# Supplementary material for: Isolation and preliminary characterization of extracellular vesicles from bottlenose dolphin (Tursiops truncatus) and long-finned pilot whale (Globicephala melas) blow
Source: PLoS One. 2026 Jul 1;21(7):e0352853. doi: 10.1371/journal.pone.0352853 (PMC13322562; doi:10.1371/journal.pone.0352853)
Supplement: S1 Fig — (PDF) [file pone.0352853.s003.pdf]

Results on long finned pilot whale EV sample

Integrin-beta 88 kDa | CD9 24 kDa | Calnexin 90 kDa | OmpA 37 kDa

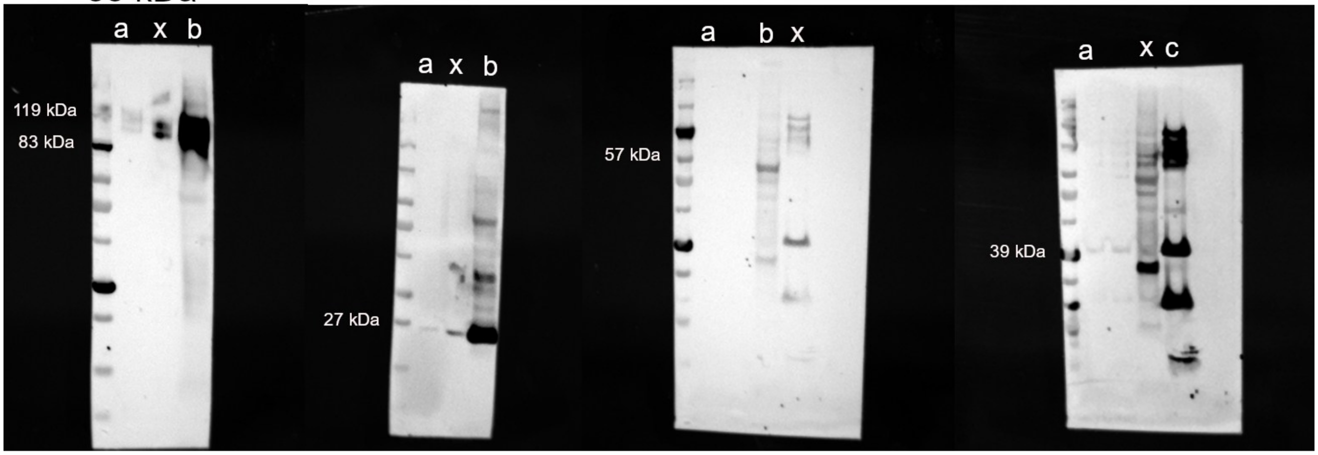

Lanes:
a: Long finned pilot whale EVs
b: Bottlenose dolphin's cells
c: E. coli lysate

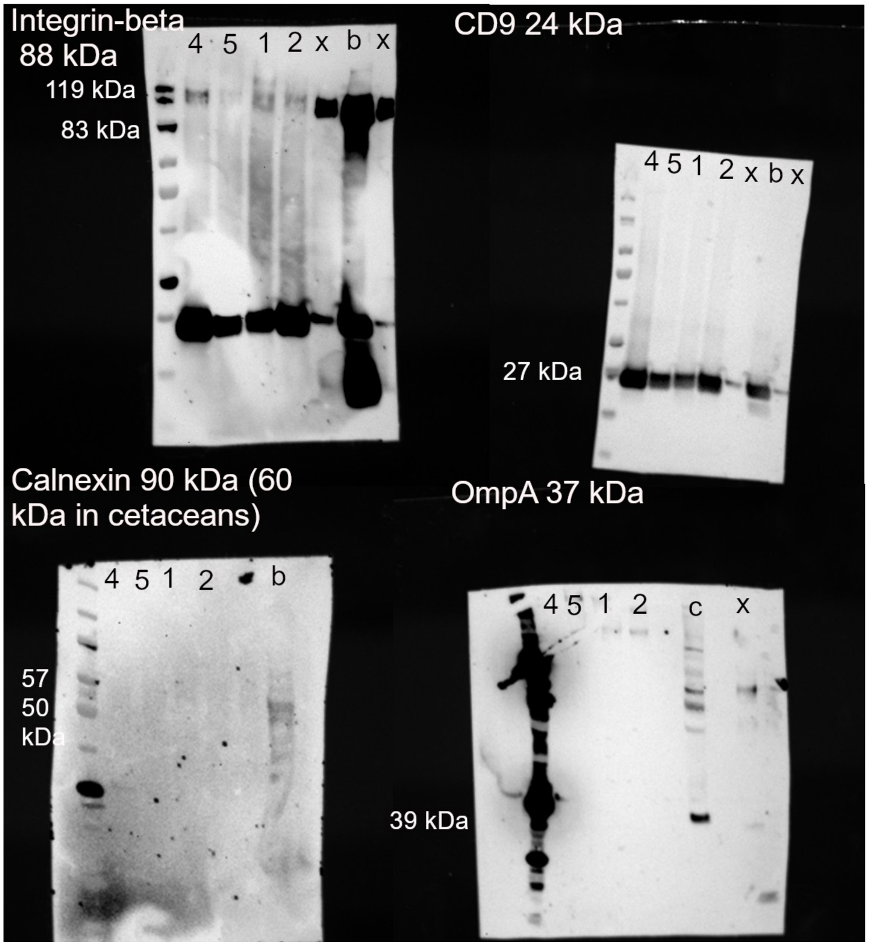

Results on dolphin UC-EVs

Lanes:
n. 1, 2, 3 ,4, 5: ID number of the dolphins included in the analysis

b: Bottlenose dolphin's cells

c: E. coli lysate

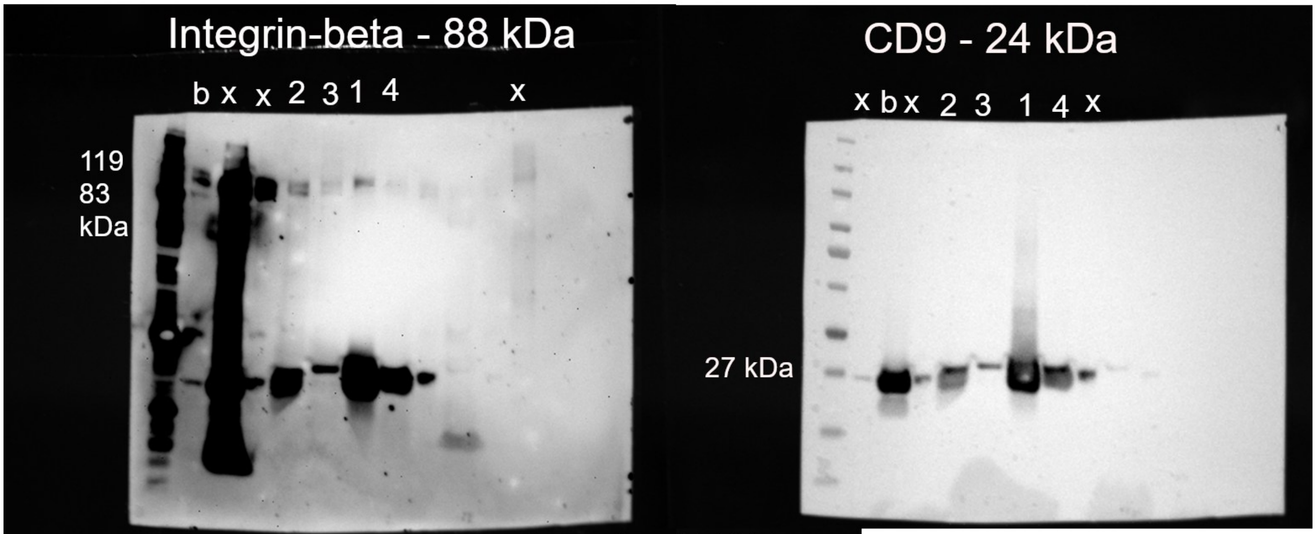

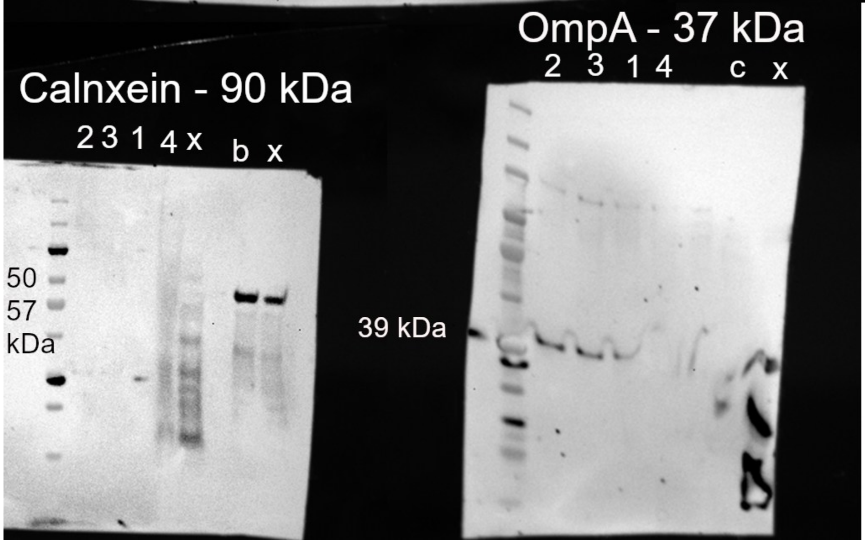

Results on dolphin SEC-EVs

Lanes:
n. 1, 2, 3, 4, 5: ID number of the dolphin included in the analysis

b: dolphin cells
c: E. coli lysate
